# Supplementary material for: Micro-optical elements from optical-quality ZIF-62 hybrid glasses by hot imprinting
Source: Nat Commun. 2024 Jun 13;15:5079. doi: 10.1038/s41467-024-49428-1 (PMC11176310; doi:10.1038/s41467-024-49428-1)
Supplement: Supplementary file 1 — Supplementary Information [file 41467_2024_49428_MOESM1_ESM.pdf]

Supplementary Information

Micro-optical elements from optical-quality ZIF-62 hybrid glasses by hot imprinting

*Oksana Smirnova[1], Roman Sajzew[1,2], Sarah Jasmin Finkelmeyer[2], Teymur Asadov[1], Sayan Chattopadhyay[1], Torsten Wieduwilt[2], Aaron Reupert[1], Martin Presselt[2,3,4], Alexander Knebel[1,3], and Lothar Wondraczek[1,3]**

[1] Friedrich Schiller University Jena, Otto Schott Institute of Materials Research, Fraunhoferstr. 6, 07743 Jena, Germany.

[2] Leibniz Institute of Photonic Technology (IPHT), Albert-Einstein-Str. 9, 07745 Jena, Germany.

[3] Friedrich Schiller University Jena, Center for Energy and Environmental Chemistry, 07743 Jena, Germany.

[4] SciClus GmbH & Co. KG, Moritz-von-Rohr-Str. 1a, 07745 Jena, Germany.

E-mail: lothar.wondraczek@uni-jena.de

This file includes:

Supplementary notes 1-3

Supplementary figures S1-S8

Supplementary notes

Supplementary note 1

Abbe number calculation

The Abbe number was calculated using the well-known equation:

$$V_D = \frac{n_D - 1}{n_F - n_C}, \quad (4)$$

where $n_D = 1.5802$, $n_F = 1.5923$ and $n_C = 1.5746$ – refractive indices of a_gZIF-62, experimentally determined by ellipsometry.

Supplementary note 2

Converting PDS absorbance to transmittance

PDS absorbance was converted to internal transmittance using Beer-Lambert law:

$$A = \log\frac{I_0}{I}, \qquad T = \frac{I}{I_0} \qquad \rightarrow \qquad T = 10^{-A} \quad (5)$$

Supplementary note 3

Focal length calculation

The lensmaker's equation was used to calculate the focal length of the imprinted lens:

$$\frac{1}{f} = (n-1)\left[\frac{1}{R_1} - \frac{1}{R_2} + \frac{(n-1)d}{nR_1R_2}\right], \quad (6)$$

where refractive index $n = 1.5802$ (determined in this work), $R_1 = -23.2$ µm (determined in this work), $R_2 \rightarrow \infty$ for a plano-concave lens, and thickness d doesn't need to be determined as the last term tends to zero.

Supplementary figures

Supplementary Fig. 1

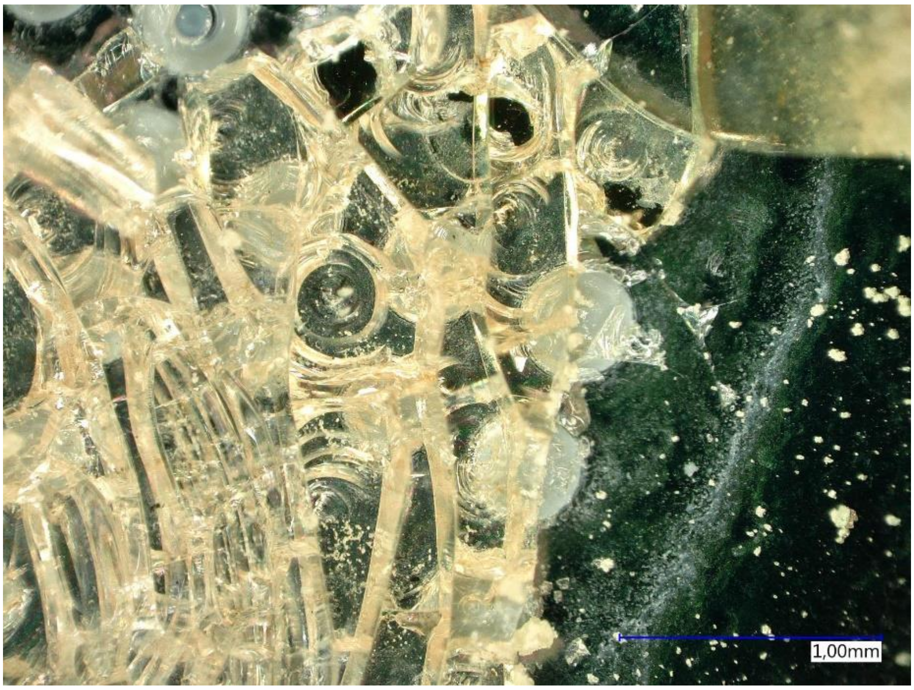

Micrograph of unsuccessful a_gZIF-62 surface micro structuring experiment. The glass has many cracks, although some thermally transferred structures are visible.

Supplementary Fig. 2

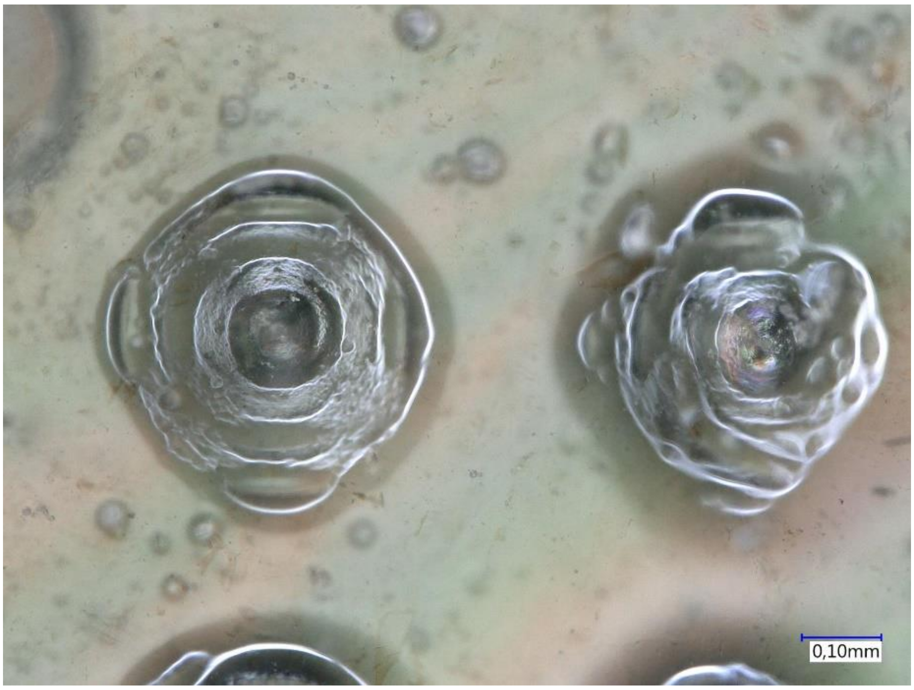

Micrograph of the first successful imprinting without cracks, produced by using the second experimental approach with test structures.

Supplementary Fig. 3

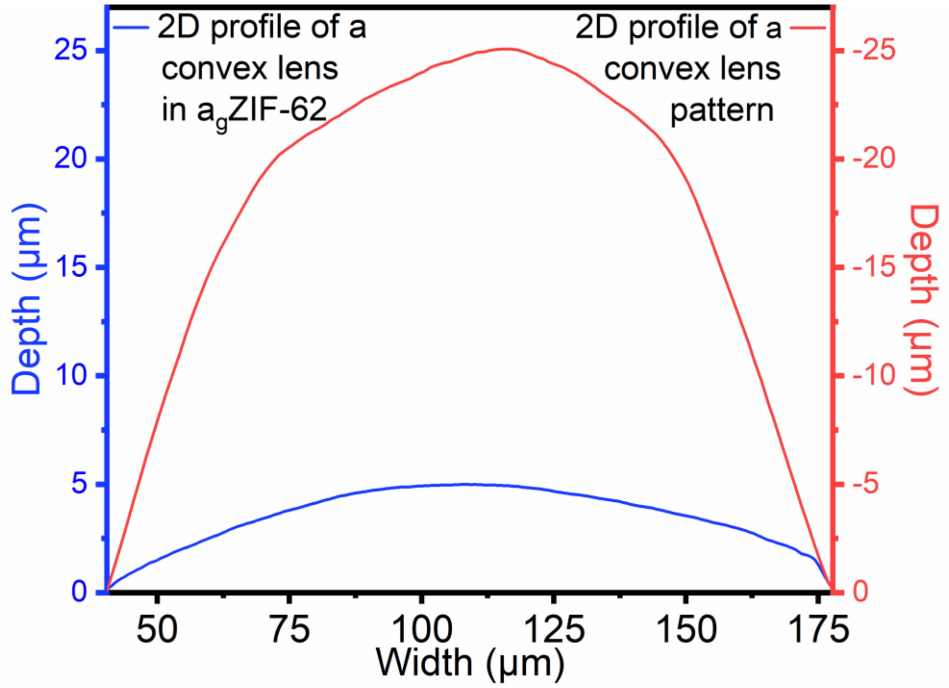

Depths profiles of the same convex lens imprint in a$_g$ZIF-62 and its template, obtained by LSM.

Supplementary Fig. 4

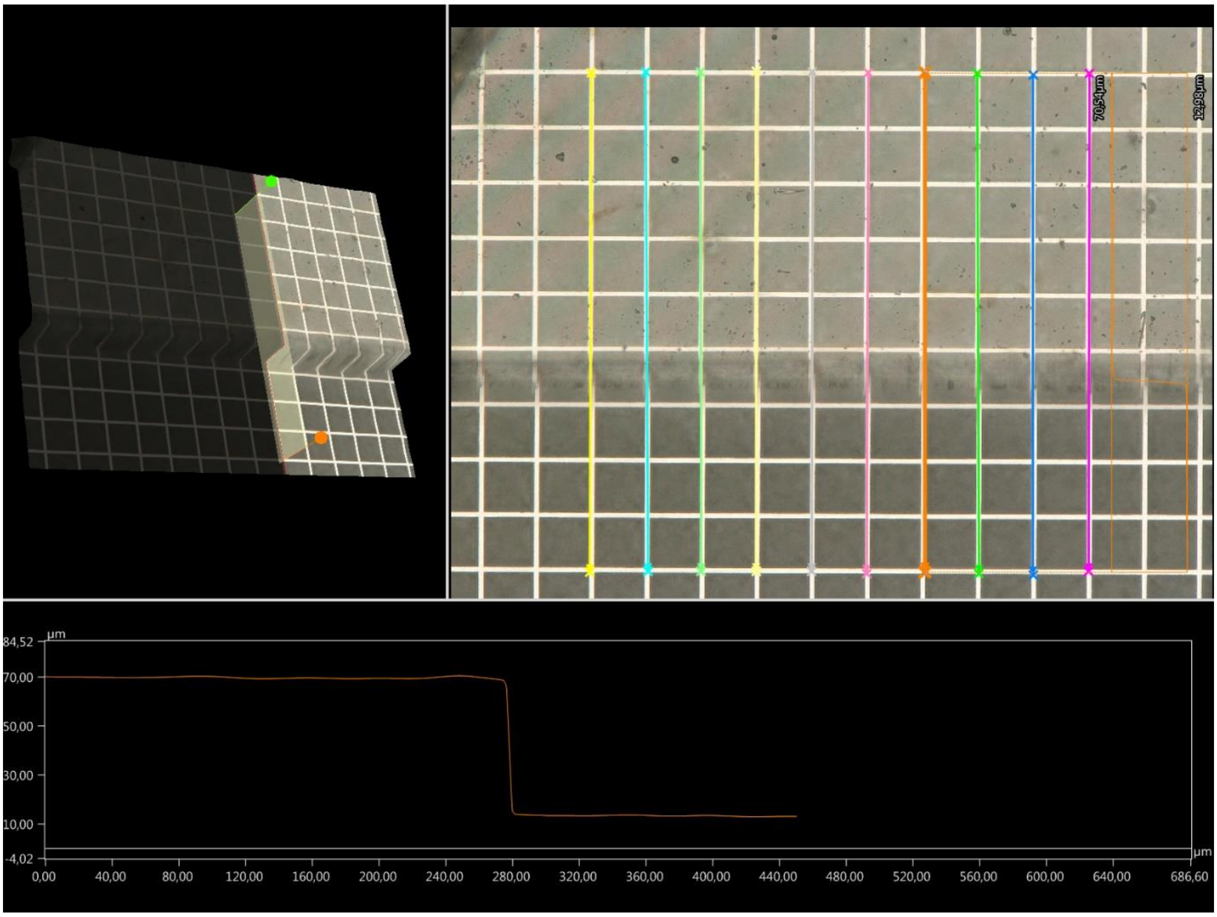

An example of dataset for optical path lenght determination collected by z-axis scanning using digital microscope: 3D image of the edge of a_gZIF-62 piece formed by z-axis scans stacking (top left), 2D profiles collected to determine the difference in depth (top right), an example of a profile (bottom).

Supplementary Fig. 5

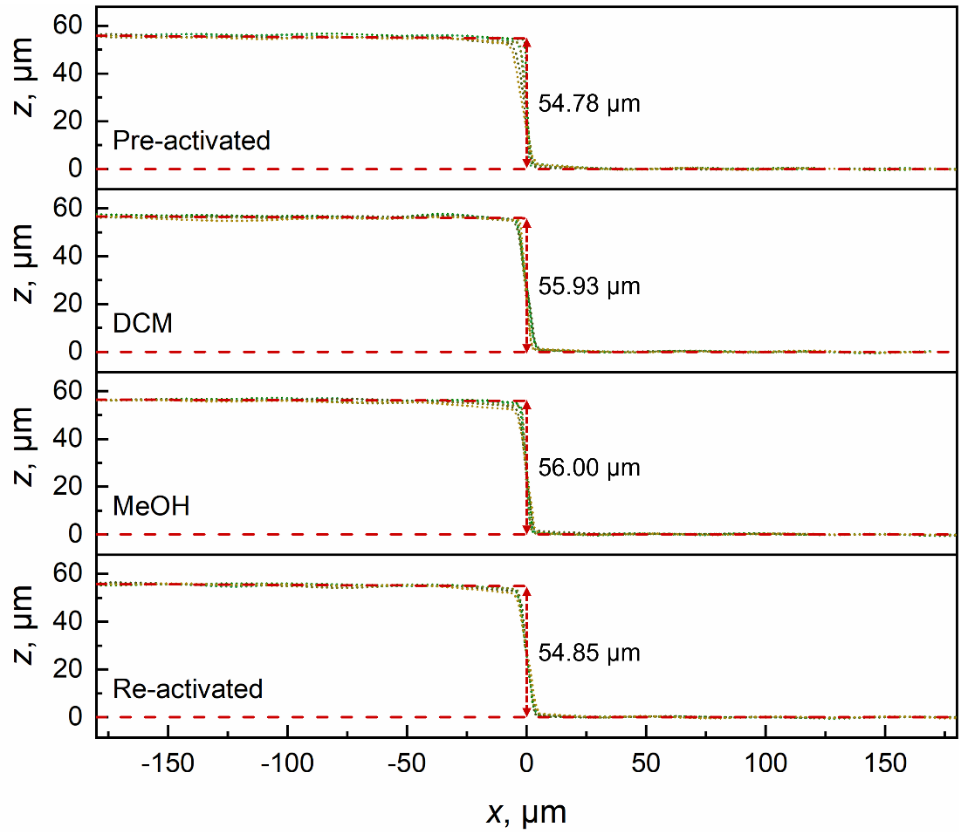

2D profiles used for the optical path length determination in pre-activated, soaked in DCM and MeOH, and re-activated a_gZIF-62 of 147.4 μm thickness; their corresponding linear approximations and calculated depths.

Supplementary Fig. 6

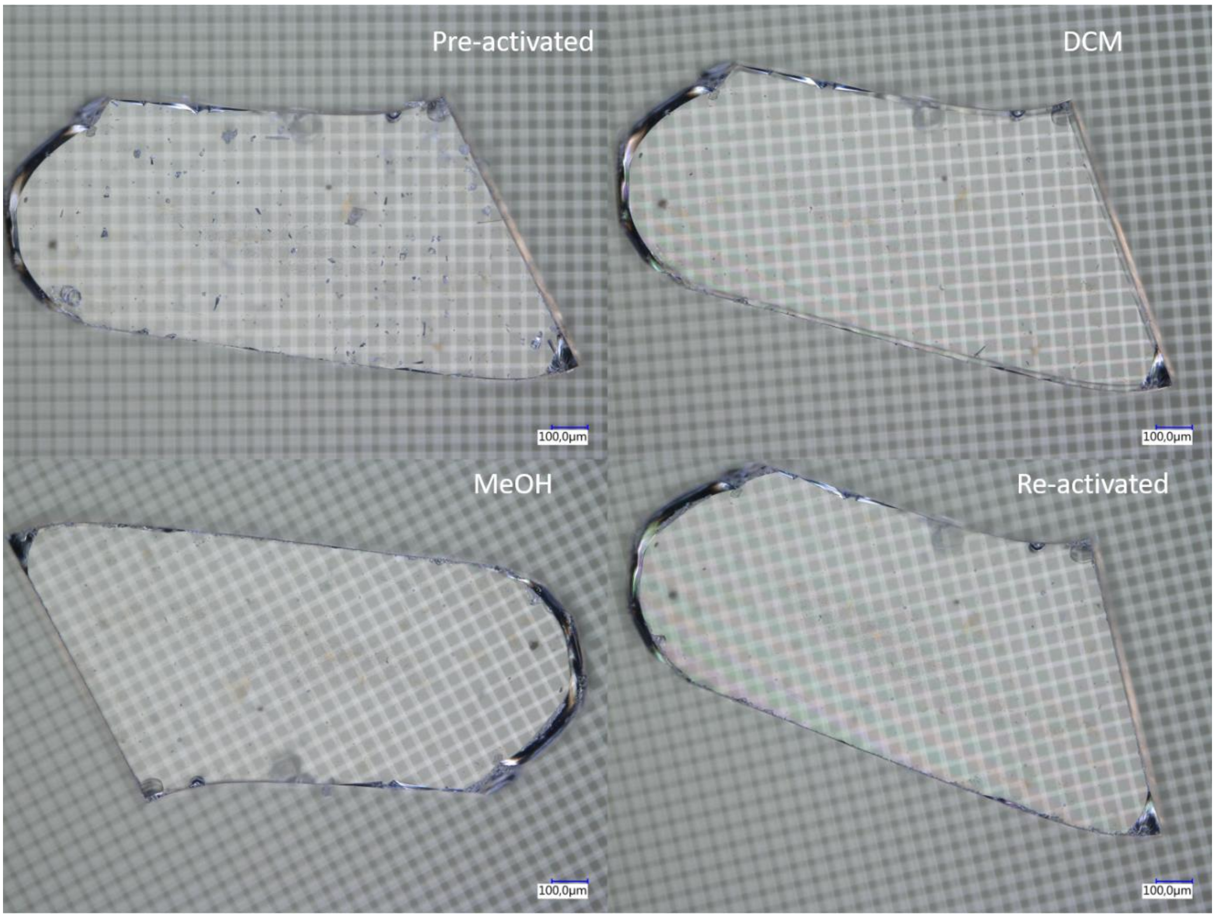

Optical photographs of the same pre-activated, soaked in DCM and MeOH, and re-activated a_gZIF-62 piece. The piece remains unchanged (besides the glass dust on the surface of pre-activated sample, which was washed away in the solvent while soaking).

Supplementary Fig. 7

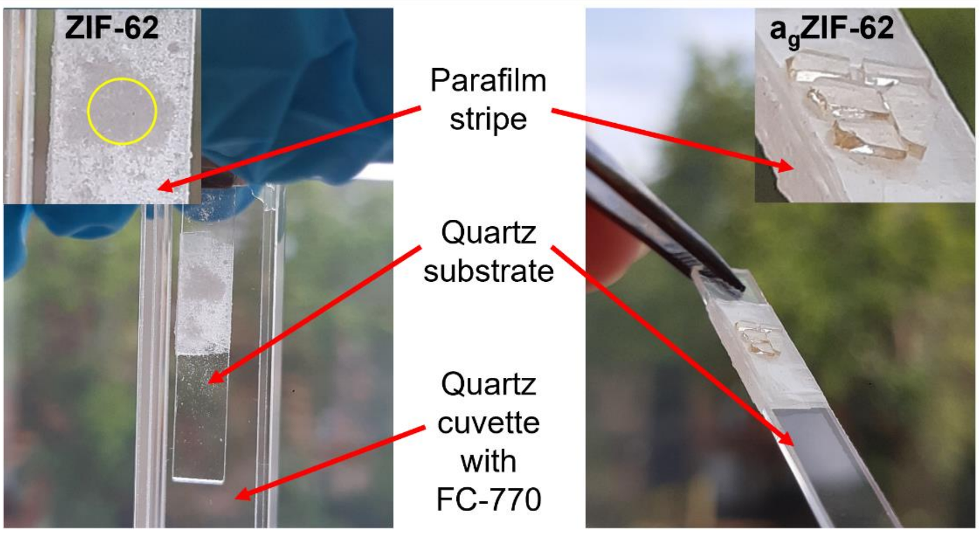

Example of sample fixture for PDS analysis.

Supplementary Fig. 8

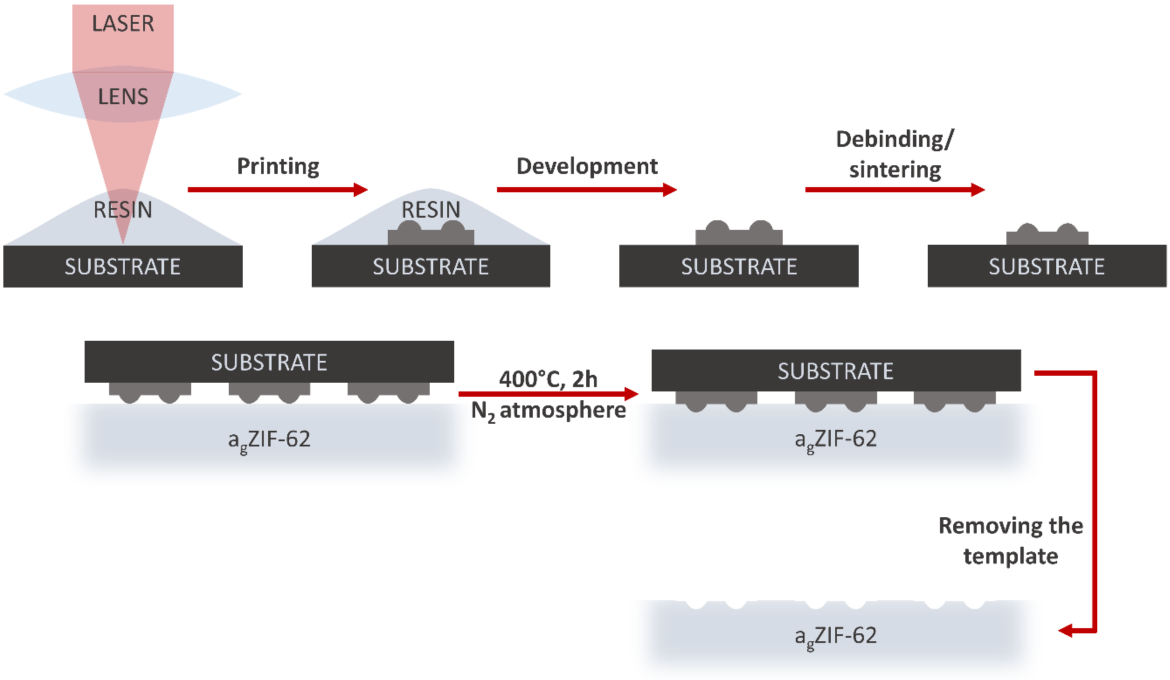

Manufacturing of the templates and hot-imprinting of micro-lenses on the surface of a_gZIF-62.
